# A body map of super-enhancers and their function in pig

**DOI:** 10.3389/fvets.2023.1239965

**Published:** 2023-10-06

**Authors:** Youbing Yang, Xinyue Li, Zhu Meng, Yongjian Liu, Kaifeng Qian, Mingxing Chu, Zhangyuan Pan

**Affiliations:** ^1^College of Animal Science and Technology, Henan University of Science and Technology, Luoyang, China; ^2^Key Laboratory of Animal Genetics and Breeding and Reproduction of Ministry of Agriculture and Rural Affairs, Institute of Animal Science, Chinese Academy of Agricultural Sciences, Beijing, China

**Keywords:** super-enhancers, pig, features, tissue-specific, function

## Abstract

**Introduction:**

Super-enhancers (SEs) are clusters of enhancers that act synergistically to drive the high-level expression of genes involved in cell identity and function. Although SEs have been extensively investigated in humans and mice, they have not been well characterized in pigs.

**Methods:**

Here, we identified 42,380 SEs in 14 pig tissues using chromatin immunoprecipitation sequencing, and statistics of its overall situation, studied the composition and characteristics of SE, and explored the influence of SEs characteristics on gene expression.

**Results:**

We observed that approximately 40% of normal enhancers (NEs) form SEs. Compared to NEs, we found that SEs were more likely to be enriched with an activated enhancer and show activated functions. Interestingly, SEs showed X chromosome depletion and short interspersed nuclear element enrichment, implying that SEs play an important role in sex traits and repeat evolution. Additionally, SE-associated genes exhibited higher expression levels and stronger conservation than NE-associated genes. However, genes with the largest SEs had higher expression levels than those with the smallest SEs, indicating that SE size may influence gene expression. Moreover, we observed a negative correlation between SE gene distance and gene expression, indicating that the proximity of SEs can affect gene activity. Gene ontology enrichment and motif analysis revealed that SEs have strong tissue-specific activity. For example, the *CORO2B* gene with a brain-specific SE shows strong brain-specific expression, and the phenylalanine hydroxylase gene with liver-specific SEs shows strong liver-specific expression.

**Discussion:**

In this study, we illustrated a body map of SEs and explored their functions in pigs, providing information on the composition and tissue-specific patterns of SEs. This study can serve as a valuable resource of gene regulatory and comparative analyses to the scientific community and provides a theoretical reference for genetic control mechanisms of important traits in pigs.

## 1. Introduction

Pigs are important livestock animals, and pork is the most widely consumed meat in Asia and Europe (1). Pigs also are valuable biomedical models for studying human diseases, pathogen responses, xenotransplantation, and drug development (2–4). To understand the genetic underpinnings of the complex characteristics and disease phenotypes in pigs and their potential applications in medical research, it is crucial to systematically annotate and functionally elucidate the regulatory elements in non-coding genomes (5).

Enhancers are distal regulatory elements that control cell type-specific gene expression (6, 7). Enhancers contain a cluster of binding sites for sequence-specific transcription factors (TFs) and co-activators, which can be several hundreds to thousands of base pairs (bp) in length (8, 9). Enhancers play a key role in controlling transcriptional programs that regulate development, cell identity, and evolutionary processes by increasing the transcription of specific target genes (10). Enhancers are often flanked by histone modifications, such as histone H3 lysine 4 monomethylation (H3K4me1) and H3K27 acetylation (H3K27ac) (11). Therefore, histone modifications serve as beacons for the detection of potential enhancers. High ratios of H3K4me1 to histone H3 lysine 4 trimethylation have been used to predict probable enhancers (12). H3K27ac is a well-characterized histone marker associated with enhanced activity (13, 14). Supported by advances in next-generation sequencing technologies, chromatin immunoprecipitation followed by sequencing (ChIP-seq) has revealed approximately one million putative enhancers in the human genome across tissues (15). These findings have sparked considerable interest in the functional and characteristic analyses of these putative enhancers. The different features and functions of enhancers in mice and humans have been thoroughly studied (16–19). Genome-wide association studies have revealed that the majority of disease-associated variations in non-coding regulatory DNA, particularly in regions enriched in enhancers (20–22). Various human diseases, including polydactyly and cancer, are affected by enhancer failure caused by genetic, structural, and epigenetic processes (19, 23, 24).

Super-enhancers (SEs) are a distinct category of enhancers (25) that have a high TF density and large size compared to typical enhancers (26, 27). SEs are large enhancer clusters filled with key TFs, cofactors, histone modification markers, and chromatin modification molecules (28, 29). SEs have a stronger ability to drive target gene transcription than typical enhancers (29) and play an important role in the maintenance of cellular properties, determination of cell fate, and disease occurrence (25, 30, 31). For instance, SEs maintain stem cell pluripotency in mammals, and their disruption causes a loss of stem cell pluripotency (32, 33). Additionally, functional analysis of the Vsx2 SE has revealed the distinctive roles of the Vsx2 enhancer components in promoting proliferation and cell fate specification during retinal development (34). Recent studies have revealed that SEs are frequently responsible for the continuous and robust transcription of oncogenes in cancer cells, such as those that cause melanoma, esophageal cancer, gastric cancer, hepatocellular carcinoma, and colorectal cancer (35–37). In addition to cancer, several studies have demonstrated that SEs play crucial roles in various disorders (38, 39). For instance, single-nucleotide polymorphisms (SNPs) linked to systemic lupus erythematosus are enriched in SEs found in B cells (30). Moreover, SEs are linked to hereditary risk factors for complicated diseases such as type 2 diabetes and coronary artery disease (40, 41). SEs have been widely identified and characterized in human and mouse genomes (42).

The genetic background of pig traits may be useful for annotating human enhancers and diseases (43). The SEs of some tissues (e.g., liver, brain, muscle, and adipose) have been reported in previous studies (44–47). To advance our understanding of SEs and their possible functions in pigs, we systematically identified SEs in 14 pig tissues by integrating ChIP-seq data labeled with H3K27ac signals and five types of enhancer annotations (48). Additionally, we analyzed the features, gene expression, gene ontology (GO) enrichment, and tissue specificity of SEs. Our study provides a map of SEs in pigs and reveals their potential functions in the regulation of tissue-specific gene expression. These results provide support for the interpretation of the biological functions of specific tissues and the analysis of genetic control mechanisms in pigs.

## 2. Materials and methods

### 2.1. Public data resources

H3K27ac and H3K4me3 ChIP-seq raw data were downloaded from a paper by Pan et al. (49) for fourteen pig tissues, including adipose, cecum, cerebellum, colon, cortex, duodenum, hypothalamus, ileum, Jejunum, liver, lung, muscle, spleen, and stomach. The data processing method was the same as the study in Pan et al. (49). All work presented in this study was based on susScr11 reference genome.

### 2.2. Identification of super-enhancers

Enhancers were classified into five categories: strong active enhancers (EnhA), methylation active enhancers with ATAC (EnhAMe), weak active enhancers (EnhAWk), activate enhancer on heterochromatic (EnhAHet), and poised enhancers (EnhPois) (Supplementary Figure S1) (50). In each tissue, the five kinds of enhancers which contain all the enhancers have been combined in a gff file. Then SEs were identified using ROSE (v1.3.1) (48) with default parameters based on the H3K27ac signals of each sample in each tissue. Next, SEs were overlapped among samples within the same tissue. A total of 8,173 non-redundant of SEs were detected after combining and merging (at least overlap of 10 K bp) them across all tissues. Tissue-specific SEs was then detected using the same approach as above for tissue-specific regulatory elements. The clustering of SEs was conducted using *k*-means (*n* = 10) in ComplexHeatmap (v.2.9.3) (51).

### 2.3. Enrichment of states in pig super-enhancers

The enrichment of states in pig SEs was assessed by (*C*/*A*)/(*B*/*D*) (52), where *A* was the size of segments in a state, B was the size of segments in a genomic feature, C is the size of segments in the overlapped region of the state and the genomic feature, D was the size of segments in the entire genome. The overlapped region used intersect function in BEDTools (v2.26.0) (53). The genomic features also included X chromosome and repeat sequences. The repeat sequences were identified using RepeatMasker (v.4.0.8) (54). And we chose this sequence content originates from retrotransposition of SINE (short interspersed nuclear element), LINE (long interspersed nuclear element) and LTR (long terminal repeat) transposable element superfamilies, as well as direct transposition of genomic DNA (55, 56).


foldenrichment=C/AB/D

### 2.4. Factors affecting gene expression

The SEs expression, tau and conservation were compared difference between genes with and without super-enhancers. The gene conservation score was sequence identity (%) from pig gene to the orthologous huma gene. The non-redundant 8,173 SEs were arranged from small to large in size and evenly divided into five groups including G1, G2, G3, G4, and G5 (Supplementary Figure S2A), then used intersect function in BEDTools (v2.26.0) (53) with parameter of -w to get the gene corresponding and expression levels of SEs. The distance between the SEs and the transcription start site (TSS) were obtained using BEDTools closest (53) with parameter of -D (Supplementary Figure S2B). Next, they were sorted from small to large and divided into five groups: two groups of downstream T1 (*n* = 4,225) and T2 (*n* = 1,982), one group of T3 (*n* = 1,000) with the distance of 0, and two groups of upstream T4 (*n* = 2,688) and T5 (*n* = 4,244), and obtained the gene corresponding and expression levels. And the T3 was used as the reference group to compare the effect of distance on gene expression. The gene expression was expressed as the mean Transcript per million (TPM) of the gene in 14 tissues.

### 2.5. GO enrichment and motif analysis

The Gene Ontology (GO) enrichment analysis of genes with SEs was performed using WebGestalt.^1^ The motif analysis and annotation used Homer’s findMotifsGenome (v.4.11) (30) with FDR < 0.05.

## 3. Results

### 3.1. Identification and summary of pig SEs

**Figure 1:**
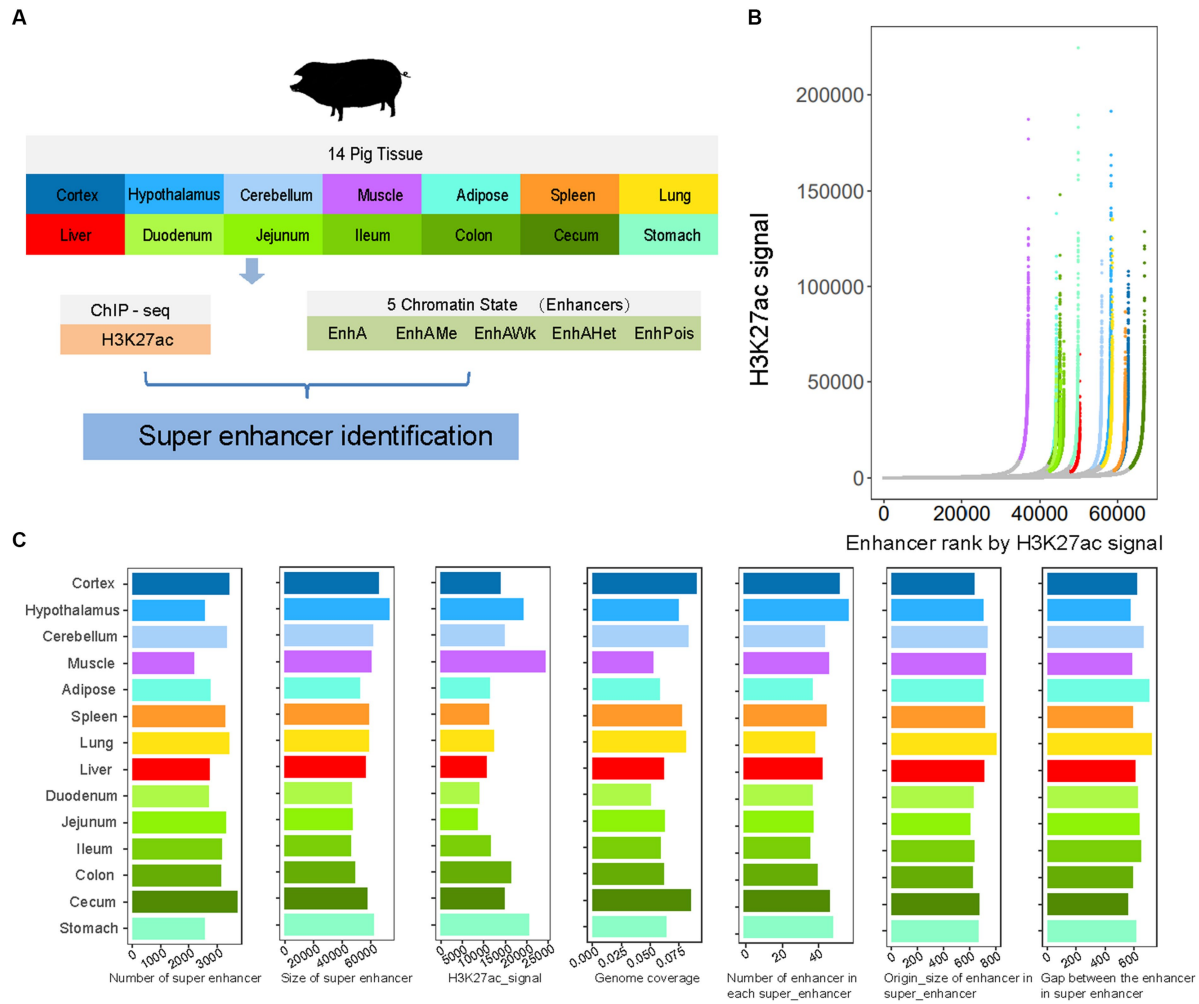
The summary of super-enhancers in pig. **(A)** The overview of the current study design. **(B)** Super-enhancers (SEs) identified by ROSE based on H3K27ac signal in each tissue. Super-enhancers are colored based on tissue, and show exceptionally high signal. Tissue color is the same with **(A)**. **(C)** The distribution of number, size, H3K27ac signal intensity and genome coverage of super-enhancers, and the number of enhancers, origin size of enhancers and gap between enhancers based on super-enhancers of 14 tissues.

SEs are generally defined as a class of regulatory regions with an unusually strong enrichment of transcription coactivator binding that play a biological role in controlling gene expression programs in tissues (transcriptional regulation, including health and disease) (48, 57). In this study, we investigated the features of SEs in 14 tissue samples (Figure 1A). We identified an average of 3,027 SEs across tissues, ranging from 2,193 in the muscle to 3,712 in the cecum, by integrating H3K27ac signals and enhancers (all five types of enhancers) (Figure 1B). The SEs had an average size of 56,862 bp, covering 42% of all enhancers and 6.85% of the pig genome. Moreover, each SE had a median of 43 enhancers and an average size of approximately 682 bp, with an average gap of 625 bp between adjacent enhancers (Figure 1C).

### 3.2. Characteristics of SEs in pigs

**Figure 2:**
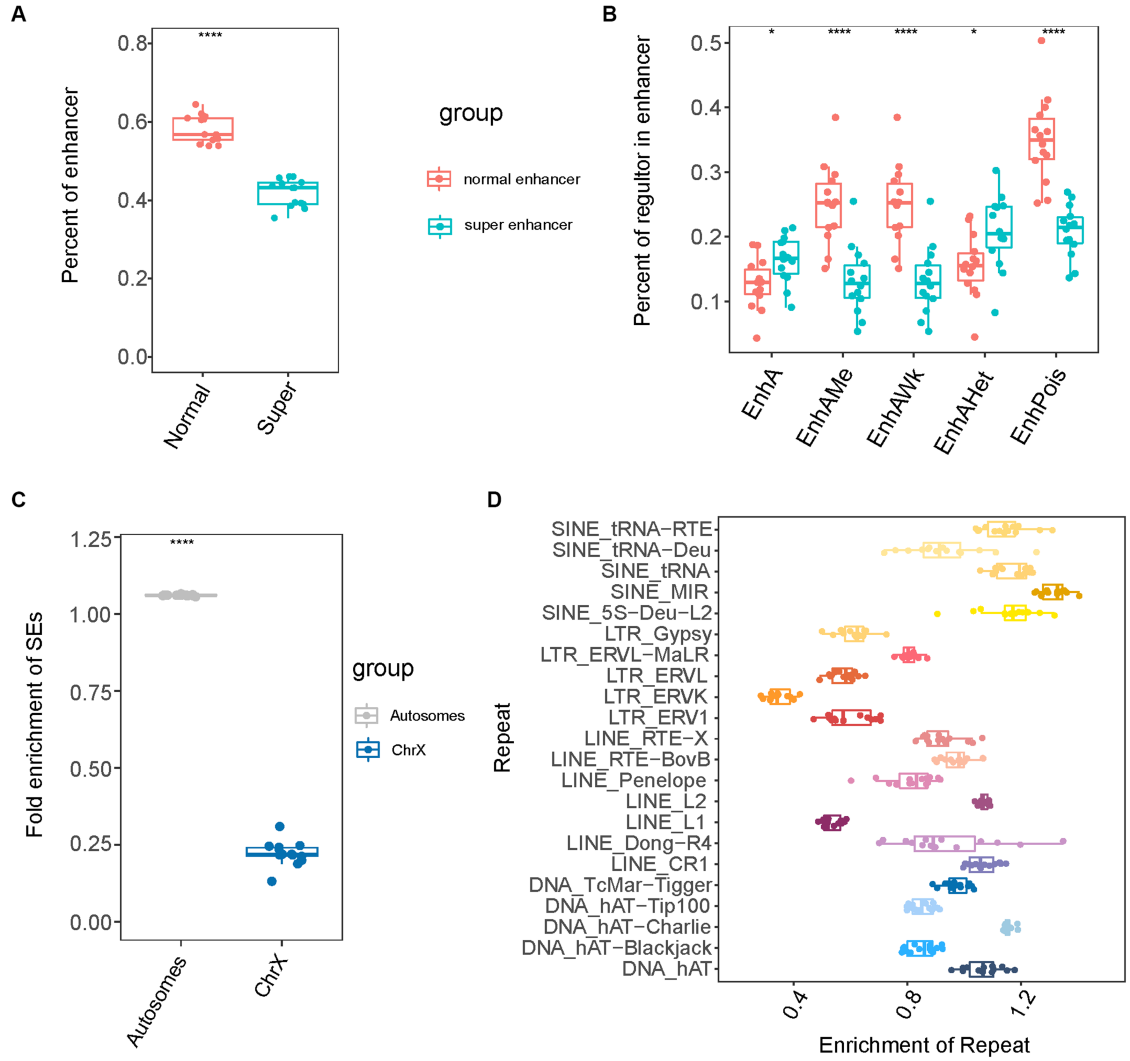
The composition of super-enhancers in pig. **(A)** The percent of enhancer in normal-enhancers and super-enhancers. **(B)** The percent of each of five enhancer types in super-enhancers and normal enhancers. **(C)** The fold enrichment of super-enhancers in autosomes and X chromosome. **(D)** The fold enrichment of super-enhancers in four repeat sequences types.

To further explore the features of SEs, we analyzed composition of the identified SEs. The SEs were large domains containing clusters of constituent enhancers (e.g., EnhA, EnhAMe, EnhAWk, EnhAHet, and EnhPois). Concerning the relationship between SEs and normal enhancers (NEs), we observed that approximately 40% of normal enhancers (NEs) formed SEs (Figure 2A). It was noted that among the five types of enhancers, SEs had many EnhA and EnhAHet, and fewer EnhPoIs than the other four types of enhancers (Figure 2B). Subsequently, we investigated the chromosomal distribution of SEs by calculating and comparing the fold enrichment of SEs on the autosomes and X chromosomes. The results showed that the fold enrichment of SEs on autosomes was approximately 1.06, whereas that on the X chromosome was only 0.2 (Figure 2C). The findings the extensive depletion of SEs in the X chromosome. Furthermore, we examined the association between SEs and repeat sequences. Repeat sequences comprise a high percentage of the genomes and are involved in tissue-specific transcriptional regulation (58, 59). Among the four major classes of transposable elements, which include short interspersed nuclear elements (SINEs), long interspersed nuclear elements (LINEs), long terminal repeats (LTRs), and direct transposition of genomic DNA, we observed that SINEs, which had higher divergence values, had the highest fold enrichment of SEs compared to the other classes (Figure 2D). Furthermore, SINE/MIR sequences showed the highest fold enrichment of SEs among the repeat sequences. We speculated that there were many repeat sequences of SINE that were older than other types in the SEs of pigs (60).

### 3.3. Influence of SEs characteristics on gene expression

**Figure 3:**
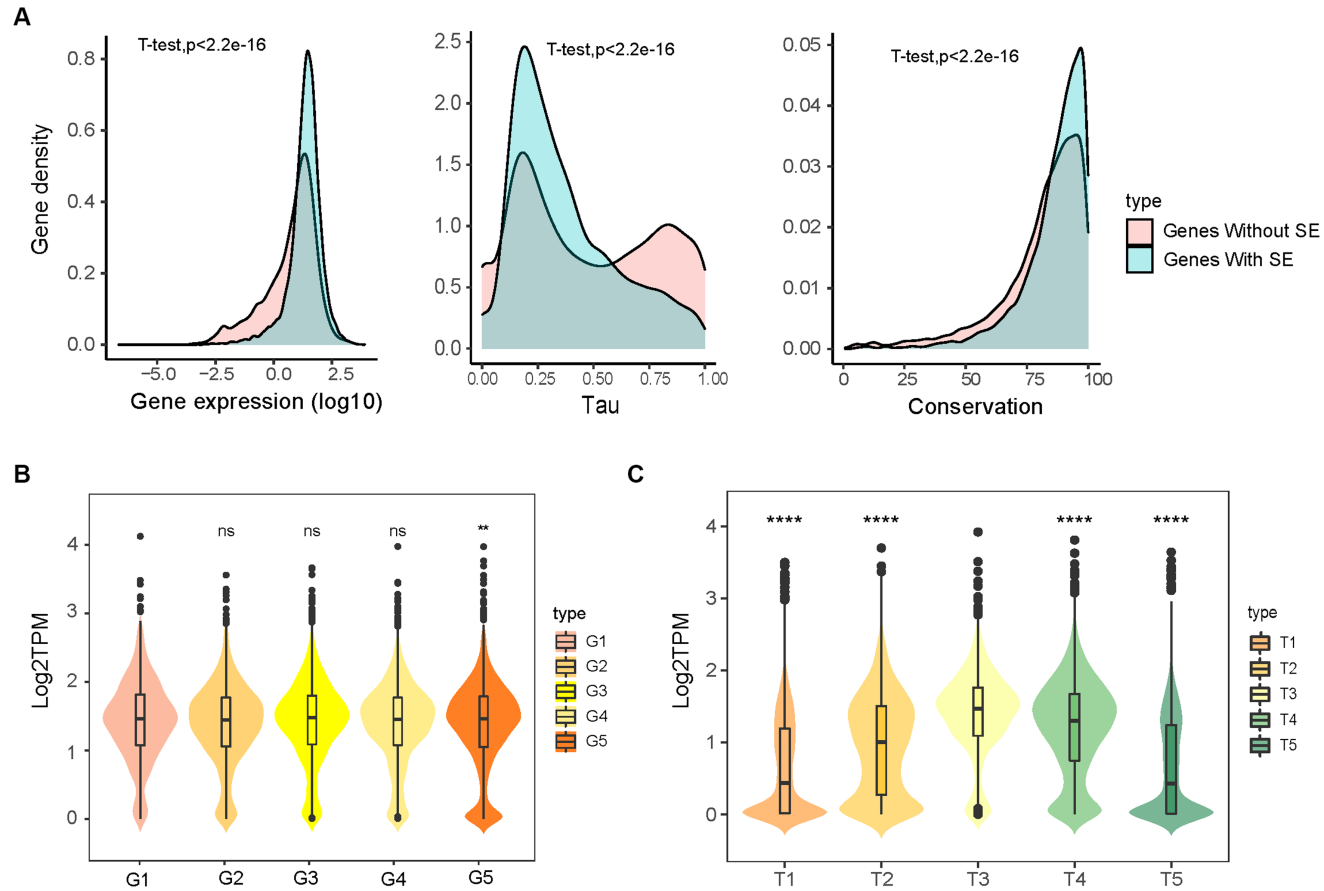
The gene expression influenced by super-enhancers. **(A)** Genes with (11,514) and without (11,886) super-enhancers differed in the expression, tau, and conservation. The gene conservation score was sequence identity (%) from pig gene to orthologous human gene. **(B)** The effect of SE length on the gene expression, categorized into five groups (G1–G5) based on increasing SE size. **(C)** The expression of genes with different distances to SEs, classified into five groups (T1–T5) based on ascending distance values: two groups of downstream genes T1 (*n* = 4,225) and T2 (*n* = 1,982), one group of genes overlapping with SEs T3 (*n* = 1,000), and two groups of upstream genes T4 (*n* = 2,688) and T5 (*n* = 4,244).

To investigate the effects of SE characteristics on gene expression, we compared the expression of SEs and NEs, SEs of different lengths, and SEs at different distances to TSS. Genes regulated by SEs showed significantly higher expression, were less tissue-specific (tau), and were more conserved than those regulated by NEs (Figure 3A and Supplementary Figure S3). This finding was in agreement with previous findings in humans and chickens (50, 61). The estimation of SE size revealed that gene expression regulated by the longest SE group was significantly higher than the shortest group (Figure 3B). Furthermore, a negative correlation was evident between the proximity of SEs to their TSS and the expression levels of the target genes (Figure 3C). Gene expression in the groups with longer (i.e., T1, T5) distances to SEs was significantly lower than that in the groups with shorter (i.e., T2, T4) distances. These results suggest that SEs features influence gene expression.

### 3.4. Tissue-specific pattern of SEs

**Figure 4:**
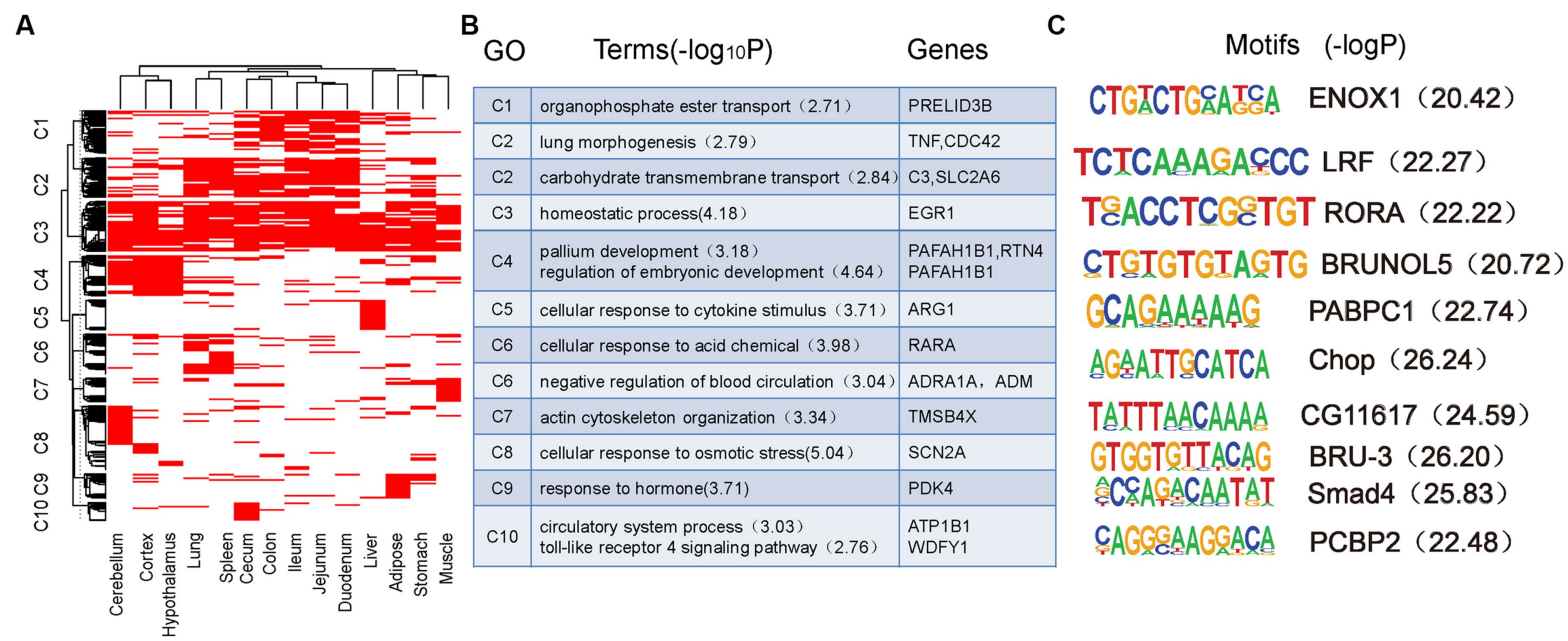
Tissue-specific pattern of super-enhancers. **(A)** Clustering of 8,173 non-redundant super-enhancers based on their activity in each tissue. **(B)** GO function and representative genes in each super-enhancers cluster. **(C)** Motifs enriched in each super-enhancers cluster.

To better understand the functions of SEs, the tissue-specific pattern of SEs was determined using GO and motif analyses. First, the SEs from the 14 tissues were merged to obtain 8,173 non-redundant SEs. Of these SEs, 27.9% (*n* = 2,281) were identified in one tissue and only 2.46% (*n* = 201) were identified in all tissues (Supplementary Figure S4). These SEs showed strong tissue-specific activity. Next, all SEs were clustered based on their activities in all 14 tissues. We found the phenomenon that some SEs were variable among tissues and revealed distinct tissue-sharing patterns for each SE cluster (Figure 4A). GO enrichment analysis of the putative target genes of these SE clusters revealed a distinct biological function of each cluster (Figure 4B). Genes targeted by SEs in C3 (Cluster 3), which are broadly active in most tissues, are involved in essential biological functions, such as homeostatic processes. SEs in C8 are specifically active in the cerebellum and their target genes are enriched in the regulation of locomotion and osmotic stress processes. SEs in C4, specific to the cerebellum, cortex, and hypothalamus, are involved in pallium development and regulation of embryonic development processes. Moreover, SEs in C1 are specifically active in intestinal tissues and correspond to phospholipid transport and glutamine family amino acid catabolic processes. SE-associated genes in C6 are specifically active in the lung and spleen and are involved in processes that include cellular responses to acid chemicals and negative regulation of blood circulation The SEs in C2 included those that were active in the lungs and spleen, in addition to those active in the intestinal tissues. The target genes of the C2 SEs are involved in lung morphogenesis, catechol-containing compound biosynthetic processes, and regulation of glucose transmembrane transport. The target genes of SEs in C5 are specifically active in the liver and were related to immune function (e.g., cellular response to cytokine stimulus). Similarly, SEs in C10 are specifically active in the cecum and are related to immune functions that include the Toll-like receptor 4 signaling pathway. SEs in C7 are specifically active in the muscle and are related to muscle activity. Finally, SEs in C9 are responsive to hormones and are specifically active in adipose tissue. Some known genes associated with SEs of each cluster are shown in Figure 4B.

Motif enrichment analysis was performed for each SE cluster to further confirm that these identified enhancers functioned as tissue-specific elements (Figure 4C). The BRUNOL5 motif enriched in C4 is associated with multiple brain diseases in humans (62). The PABPC1 motifs enriched in C5 playes a role in the liver (63). The LRF motif enriched in C2 contributes to gut and immune tissues (64). Smad4 enriched in C9 is important in adipogenic differentiation and has been described in human adipose tissue-derived stem cells (65). The CHOP motif enriched in C6 is related to pulmonary fibrosis and immune function in mice (66). The PCBP2 motif enriched in C10 also plays a role in immune function (67). Furthermore, we observed increased expression of genes with tissue-specific SEs in the corresponding tissues. These results demonstrate the importance of SEs in tissue specificity and function in pigs.

### 3.5. Tissue-specific SEs in brain and liver tissues

**Figure 5:**
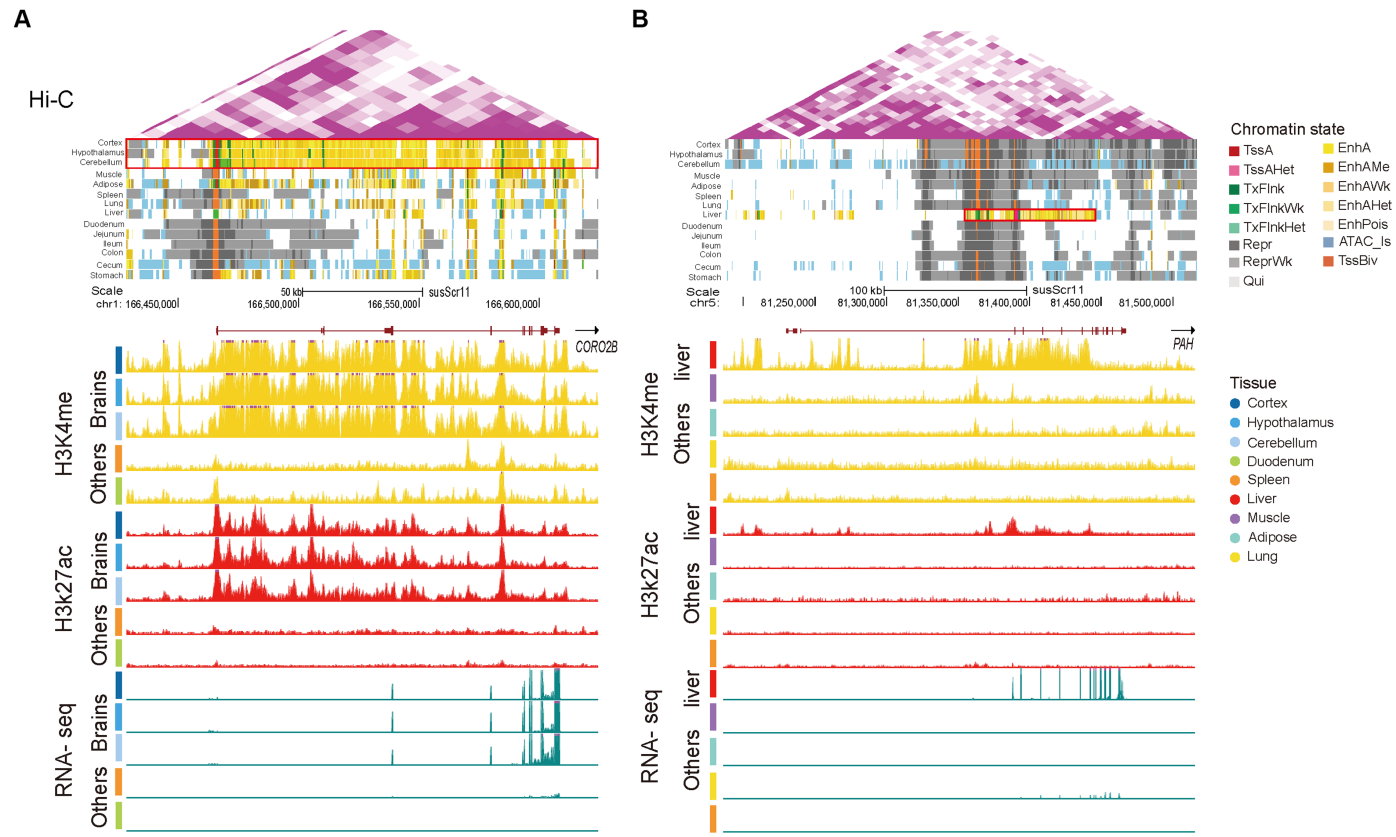
Super-enhancers in brain and liver tissues. **(A)** A brain-specific super-enhancer present at the CORO2B locus (chr1:166,239,933-167,241,533). **(B)** A liver-specific super-enhancer present at the PAH locus (chr5:81,236,096-81,453,790). Following sections are Hi-C, chromatin state, H3K4me1, H3K27ac, and RNA-seq. Vertical scale of UCSC tracks shows normalized signal from 0 to 100 for RNA-seq, 0 to 150 for H3K27ac, and 0 to 50 for H3K4me1. The red boxed areas are the range of SEs.

To further illustrate the situation of SEs, we selected the tissue-specific SEs in the brain and liver. Coronin 2B (*CORO2B*), a gene with brain-specific SEs, displayed obvious elevated H3K4me1 and H3K27ac signaling and gene expression in brain tissue (Figure 5A). This gene plays a prominent role in maintaining correct distribution of the cytoskeleton and neuronal migration (68). Moreover, the phenylalanine hydroxylase gene (*PAH*) has been linked to liver-specific SEs with strong liver-specific H3K4me1 and H3K27ac signaling (Figure 5B), showing liver-specific expression. The *PAH* gene is specifically expressed in the human liver and is associated with phenylketonuria (69, 70). Overall, these results suggest that tissue-specific SEs play important roles in the regulation of tissue-specific functions.

## 4. Discussion

SEs can be useful genetic areas for selection and breeding because selection concentrates on functional SEs (71). The pig genome sequence is publicly available, enabling the systematic discovery and characterization of SEs in this species. In the present study, we utilized ChIP-seq data labeled with H3K27ac signals and five types of enhancer annotations to identify 42,380 SEs in 14 pig tissues. We compared the features and functions of the SEs and NEs and revealed gene expression, composition, pathway enrichment, and tissue specificity of the SEs. Our findings will aid in the understanding of the regulatory environment of porcine SEs and their potential applications in genomic selection.

We explored the composition of SEs from various perspectives. Our results demonstrate that SEs are distinct from NEs in terms of the size and content of TFs and transcriptional activity. SEs were significantly larger and more likely to be enriched with the activation enhancer. Previous studies have demonstrated that contacts between the enhancers that make up these super-regulatory elements are functionally influential (72), and that individual enhancer elements within the SEs have additive or synergistic relationships (72, 73). Further studies are needed to clarify the interactions of individual enhancer elements within SEs. Moreover, we observed that SEs were seriously depleted in the X chromosome, indicating that SEs were not present on the X chromosome. This finding may be related to our data from male pigs (49). In the present study, the repeat sequences of the pig genome were mainly composed of SINEs and LINEs, followed by DNA retrotransposons and LTR transposons. This is consistent with earlier research that demonstrated a high percentage of LINE and SINE retrotransposons in repetitive sequences of mammalian genomes (74). The SINE/MIR sequences had the highest fold enrichment of SEs compared to the other repeats. This could be because SINE/MIR sequences displayed were derived from a family of ancient SINEs and are distributed among a wide range of species (60), indicating that the SEs of SINE/MIR may play important roles in species evolution.

Genes regulated by SEs were significantly more highly expressed, less tissue-specific, and more conserved than those regulated by NEs. These results are consistent with those of earlier studies in humans and chickens (50, 61). We investigated the effects of various SE features on gene expression. Our preliminary results indicated that SE length influences gene expression. According to an earlier study, the complexity of a regulatory task is correlated with an increase in enhancer length and number of TF-binding sites (75). SEs may have features similar to those of enhancers, which require further study. Additionally, we examined the relationship between the expression levels of the target genes and the distance between the SEs and the TSS. We observed that gene expression positively correlated with the proximity of SEs to TSS, indicating a proximity effect of SEs on gene regulation. Collectively, these results suggest that SE features can influence gene expression.

Clustering of all SEs based on their activities in all 14 tissues revealed that some SEs were variable among tissues and displayed distinct tissue-sharing patterns for each SE cluster. SEs are not fixed entities but are dynamic and context-dependent (76). For example, SEs are regulated genes that specify cell fate and identity by the sequential addition or removal of enhancers during differentiation (77). Therefore, the phenomenon reflects the complexity and diversity of gene regulation. GO enrichment analysis and motif analyses were performed to investigate the tissue specificity of SEs. Intestine-specific SEs were found to be involved in the circulatory system and nutrient absorption, matching the function and biology of the intestine. These SEs may play key roles in the growth and development of pigs. We also identified liver- and cecal-specific SEs that are associated with the regulation of immune function and the development and suppression of diseases such as diabetes and liver cancer (30). Pallium development is strongly correlated with the presence of brain-specific SEs. We identified a brain-specific SE in the *CORO2B* gene, which is important in neuronal migration in mice, and shows strong brain-specific expression (68, 78). Neuronal migration during cortical development is essential for maintaining normal brain function and structure. Impairments in neuronal migration can lead to various neurodevelopmental and neuropsychiatric disorders in humans, such as Timothy syndrome, schizophrenia, and autism spectrum disorders (79–81). Moreover, we found that *PAH* genes with liver-specific SEs exhibited strong liver-specific expression. The *PAH* gene codes for the enzyme phenylalanine hydroxylase (70). Pathogenic variants of the *PAH* gene can result in phenylketonuria, which causes liver dysfunction (82). The results reveal the tissue specificity of SEs and provide insights into the regulatory mechanisms of disease in pigs.

In conclusion, our analysis of SEs in the pig genome provides a body map of SEs and clarifies their features and functions. The findings will support the identification of the biological functions of particular tissues and provide a valuable resource for transcriptional regulation studies in pigs.

## Data availability statement

Publicly available datasets were analyzed in this study. This data can be found at: https://www.ncbi.nlm.nih.gov/bioproject; PRJEB37735.

## Ethics statement

Ethical approval was not required for the study involving animals in accordance with the local legislation and institutional requirements because raw data were downloaded from public databases.

## Author contributions

YY, XL, and ZP participated in the design of the study. YY and XL carried out the analysis, interpretation of data, and initiated, drafted, and revised the manuscript. ZM wrote sections of the manuscript. YL, KQ, and MC revised the manuscript. All authors contributed to the article and approved the submitted version.

## Footnotes

^1^ http://www.webgestalt.org/
