# Supplementary figures and images for: A body map of super-enhancers and their function in pig
Source: Front Vet Sci. 2023 Oct 6;10:1239965. doi: 10.3389/fvets.2023.1239965 (PMC10587440; doi:10.3389/fvets.2023.1239965)

**A**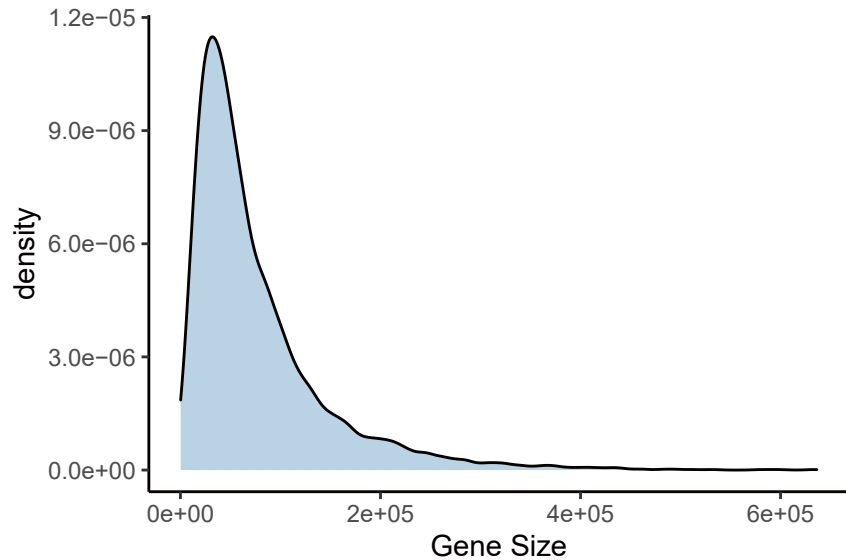**B**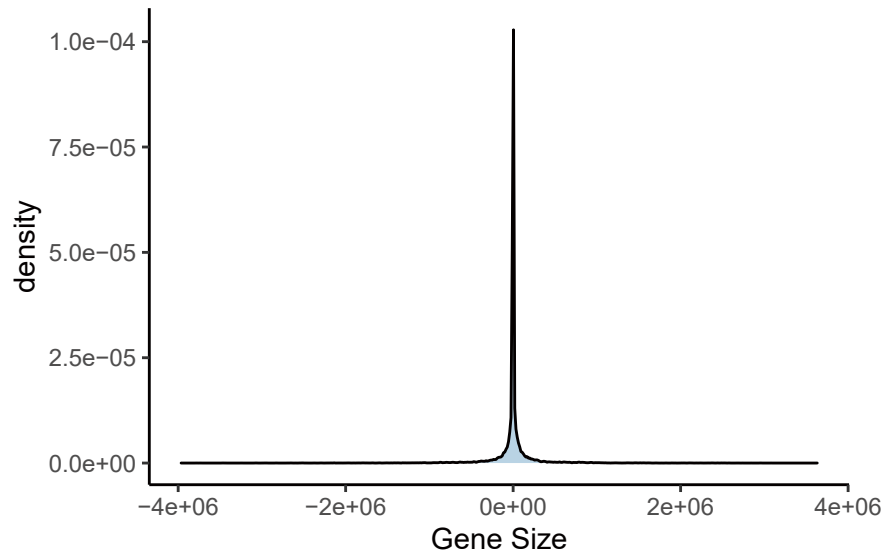

Supplement: Supplementary file 1 [file Data_Sheet_1.PDF]

Number of super enhancer

2000

1000

0

1

2

3

4

5

6

7

8

9

10

11

12

13

14

Number of tissues

1260

1004

748

602

483

449

334

361

274

272

310

251

230

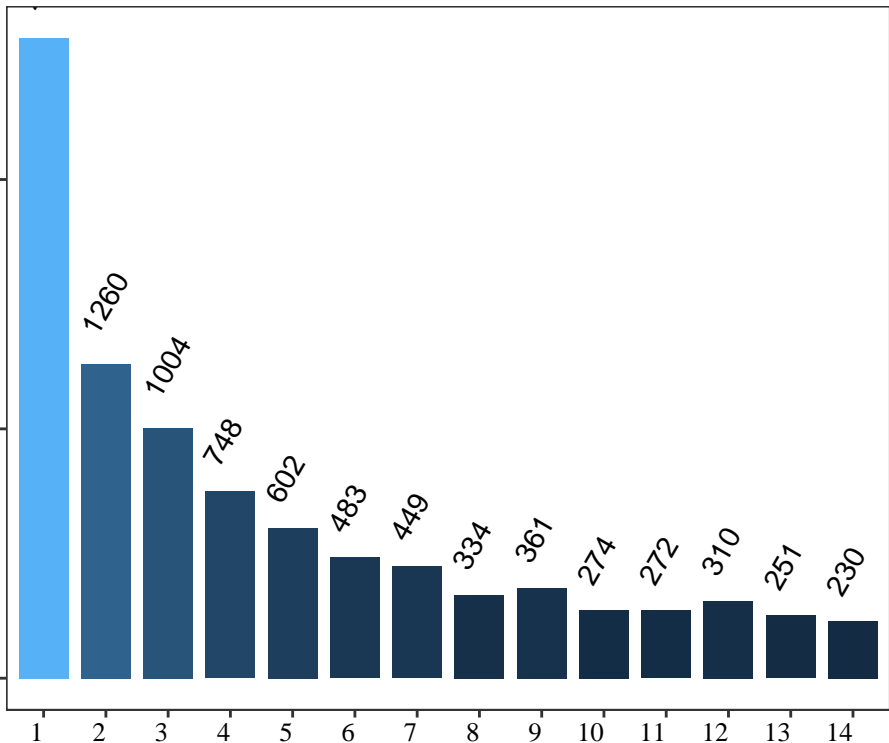

Supplement: Supplementary file 2 [file Data_Sheet_2.PDF]

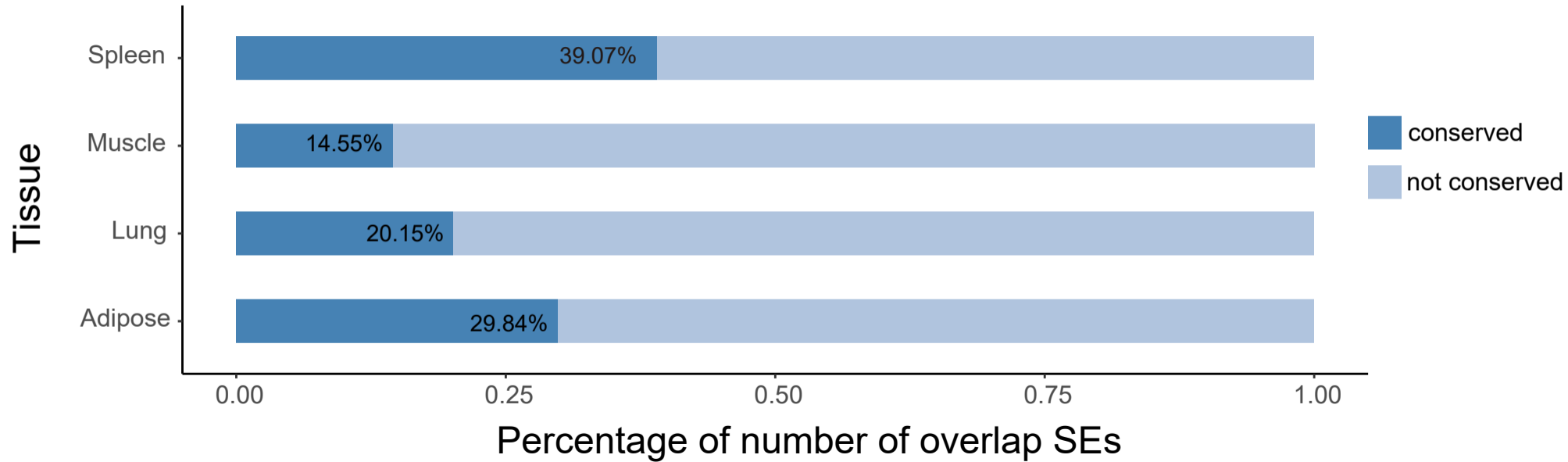

Supplement: Supplementary file 3 [file Data_Sheet_3.PDF]

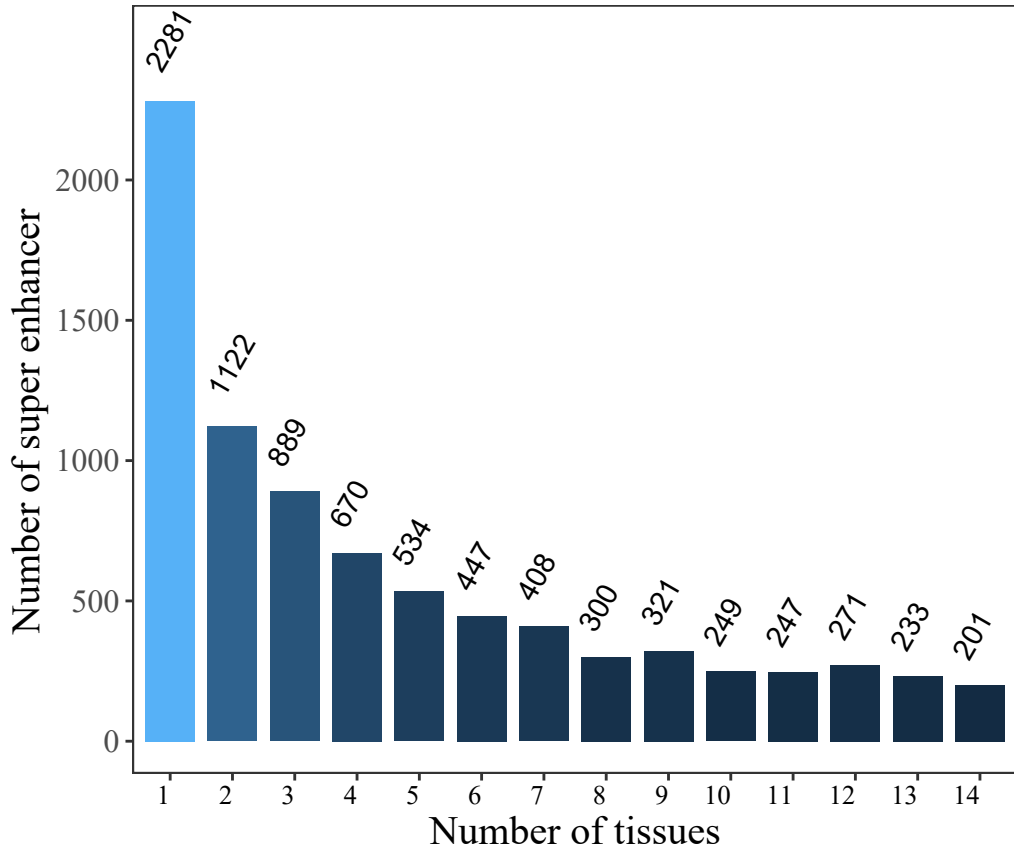

Supplement: Supplementary file 4 [file Data_Sheet_4.PDF]
